# Chemical Composition and Antimicrobial Activity of a New Olive Pomace Functional Ingredient

**DOI:** 10.3390/ph14090913

**Published:** 2021-09-10

**Authors:** Maria Antónia Nunes, Josman Dantas Palmeira, Diana Melo, Susana Machado, Joana Correia Lobo, Anabela Sílvia Guedes Costa, Rita Carneiro Alves, Helena Ferreira, Maria Beatriz Prior Pinto Oliveira

**Affiliations:** 1 REQUIMTE/LAQV, Laboratory of Bromatolgy, Department of Chemical Sciences, Faculty of Pharmacy, University of Porto, R. Jorge de Viterbo Ferreira, 228, 4050-313 Porto, Portugal; antonianunes.maria@gmail.com (M.A.N.); melo_dian@hotmail.com (D.M.); su_tche@hotmail.com (S.M.); joanaclobo@sapo.pt (J.C.L.); acosta@ff.up.pt (A.S.G.C.); beatoliv@ff.up.pt (M.B.P.P.O.); 2 REQUIMTE/UCIBIO, Laboratory of Microbiology, Department of Biological Sciences, Faculty of Pharmacy, University of Porto, R. Jorge de Viterbo Ferreira, 228, 4050-313 Porto, Portugal; josmandantasp@gmail.com (J.D.P.); hferr@ff.up.pt (H.F.); 3 Department of Biology & CESAM, Campus de Santiago, University of Aveiro, 3810-193 Aveiro, Portugal

**Keywords:** olive pomace, sustainability, antimicrobial activity, natural additive

## Abstract

Olive pomace, an olive oil processing byproduct, can be upcycled and meet the current demand for natural and sustainable food ingredients. In this work, a patented process was used to obtain a functional ingredient from different olive pomaces. The nutritional, chemical and antioxidant profiles, as well as the antimicrobial activity against *S. aureus*, *E. coli* and *C. albicans*, were investigated for the first time. The amount of phenolics ranged between 3.1 and 3.8 g gallic acid eq./100 g in all samples and flavonoids between 2.0 and 3.2 g catechin eq/100 g. No significant differences were found regarding the antioxidant activity. The total fat varied between 5 and 11%, α-tocopherol being the major vitamer and oleic acid the main fatty acid. The protein and ash contents were 1–4% and 10–17%, respectively. The functional ingredient with a higher hydroxytyrosol content (220 mg/100 g) also presented the best minimal inhibitory concentration against the tested bacteria. No activity against *C. albicans* was verified. This new functional ingredient presents the potential to be used as a natural preservative or as a nutritional profile enhancer. Moreover, it can be an advantageous ingredient in food products, since it comprises specific lipid and hydrophilic bioactive compounds usually not present in other plant extracts.

## 1. Introduction

Industrial players and research institutions have been firmly committed to the circular economy principles by exploring efficient and eco-friendly procedures to upcycle agro-industrial byproducts [1]. All phases of the food life cycle (agricultural production, processing and distribution) generate byproducts that are natural resources available in high amounts at low costs and, noticeably, can be sources of high-value nutritional components [2]. 

In 2020, the EU produced more than 1900 thousand tons of olive oil. Spain was the top producer, followed by Italy, Greece and Portugal [3]. Olive oil production is increasing due to its organoleptic properties and significant benefits in health. Furthermore, the olive oil yield is 15–20%, meaning that the remaining material is olive pomace (80–85%) [4]. Therefore, the generation of this byproduct is currently high and tends to increase. Olive pomace can be a great source of components with bioactivity, such as antioxidants and fatty acids, that can be explored to develop new ingredients or products [5,6]. It was reported that olive products (olive and olive pomace oils) and byproducts exert antimicrobial activity against pathogenic bacteria and toxigenic fungi strains [6]. This effect is mainly related to the presence of phenolics, probably in a synergistic way, and the amount and type of phenolics present can influence the bioaction efficiency [6].

The use of synthetic additives in the food industry is a recurring topic in public health discussions due to the hazard risk related to the concomitant consumers and long-term exposure [7]. This movement aligns with the current health concerns and environmental awareness, which has been guiding consumer behaviors over the last years [8]. Currently, consumers prefer products additive-free or with natural ingredients. Thus, the utilization of natural additives derived from olive oil processing byproducts can address the consumer trend, which is also a challenge for food and beverage companies [9].

This study assessed the chemical composition and antimicrobial activity against the bacteria (*Staphylococcus aureus* and *Escherichia coli*) and yeast (*Candida albicans*) of four olive pomace-based ingredients in order to evaluate their potential to be used in food products.

The olive pomace extracts were obtained using an ecological and innovative process (patent international application PCT/IB2018/060111, 2019) and are natural, plant-based and chemical-free. The functional ingredient presented antimicrobial activity against *S. aureus* and *E. coli*, foreseeing its application in food products as a natural and sustainable preservative. Additionally, it can also be valuable to improve the nutritional profile of food products due to the bioactive compounds’ health effects, especially hydroxytyrosol.

## 2. Results and Discussion

In this work, a green, sustainable and cost-effective method was used to add value to olive pomace, currently generated in high amounts in the Mediterranean basin, by recovering its bioactive compounds in a functional ingredient. Pomaces from the two major Portuguese olive oil production regions—Trás-Os-Montes (O1 and O2) and Alentejo (O3 and O4)—were analyzed. Table 1 shows the geographical origin and the predominant varieties in each pomace.

The phytochemicals (total phenolics and flavonoids) and antioxidant activity results are summarized in Table 2. Different methods were also applied to evaluate the antimicrobial activity of the olive pomace extracts. The incorporation, surface spreading and disk diffusion methods were used for screening purposes, whereas the microdilution method assessed the MIC. A Gram-positive and a Gram-negative bacteria and yeast (*S. aureus*, *E. coli* and *C. albicans*, respectively) were selected as representatives of the principal types of food contamination and human infection (Table 3 and Table 4). Table 3 summarizes the preliminary results for the extracts’ antimicrobial activity, being clear that the extracts had an antimicrobial activity for *E. coli* and *S. aureus* but not for *C. albicans*. Table 4 shows the results of the antimicrobial activity against *E. coli* and *S. aureus*.

Regarding the antimicrobial action, the most studied natural compounds are phenolics, although the action of oligopeptides, terpenoids or macrolides have also been reported [10,11,12,13]. 

Phenolics are secondary plant metabolites, able to act as antioxidants by neutralizing reactive oxygen and nitrogen species due to hydroxyl groups, making them good hydrogen donors. Furthermore, they can moderate free radical generation by chelating metal ions. Its antimicrobial action depends on the respective concentration and chemical structure [11]. These compounds can cause the disruption of the membrane structure and leakage of the intracellular components caused by phenolics hydroxyl (–OH) groups interacting with bacteria membranes [14]. The antibacterial (both Gram-positive and Gram-negative) effect that has been most assessed, and attributed to phenolics, is membrane disruption. The –OH group in phenolics leads to electron delocalization, making them act as proton exchangers. This will promote a gradient reduction across the cytoplasmic membrane of bacterial cells, leading, ultimately, to the cell’s death [11].

O1 and O3 presented the highest amounts of phenolics (3.5 and 3.8 g GAE/100 g, respectively) and O3 the highest flavonoids content (3.2 g CE/100 g) (Table 5).

Simultaneously, O1 presented the highest content of hydroxytyrosol (220 mg/100 g) and the best minimal inhibitory concentration for *E. coli* (62.5 mg/mL) (Table 2 and Table 4). This value was half the value presented by the other extracts (125 mg/mL). In the case of *S. aureus*, both O1 and O3 presented a similar MIC (31.25 mg/mL) and the lowest value compared to the other samples (Table 4). The O4 MIC value (125 mg/mL) was the highest related to the lowest content in hydroxytyrosol, and O3 presented intermediate values for these two parameters in discussion (Table 2 and Table 4).

O3 presented the highest amount in total flavonoids (3.2 g CE/100 g), a phenolics class. This sample also showed the best MIC result, along with O1, against *S. aureus*. The flavonoids described in olive pomace include quercetin, apigenin, rutin, luteolin glucoside, luteolin, dismetin, taxifolin and apigenin glucoside [15]. The flavonoids’ molecular actions are mainly due to their capacity to form complexes with proteins that compromise microbial adhesion, microbial enzymes and cell transport proteins, among other mechanisms. Overall, fundamental mechanisms have been proposed for flavonoids: damage of the cytoplasmic membrane, topoisomerase and NADH-cytochrome c reductase inhibitions [16]. Table 6 lists the proposed antimicrobial mechanisms of the phenolics and flavonoids.

Regarding the antioxidant activity evaluated by the DPPH assay, O1 showed the lowest value (0.6 g TE/100 g), although with no significant differences when compared to O4 (0.7 g TE/100 g). The same sample presented the highest value in the ABTS assay, along with O3 (1.13 and 1.1 g ET/100 g, respectively), parallel to the hydroxytyrosol values of these samples. 

These data can indicate that other compounds present in the extracts beyond the ones analyzed can have antioxidant actions. 

The selected olive pomace samples are from distinct geographical origins and have different predominant olive cultivars (Table 1). Furthermore, olive processing can influence their phytochemicals amount and profile: modifications not only occur in the amount of phenolics of olive paste, oil and byproducts (due to transference among the fractions) but, also, in the phenolics profile, indicating transformations at the compound level. The olive secoiridoids, such as oleuropein, demethyloleuropein and ligstroside, are degraded during crushing and malaxation, the olive processing initial stages, forming several secoiridoid aglycone derivatives. Despite not being detected in the fruits, tyrosol and hydroxytyrosol and its glucosides occur in all the products of olive oil processing. Only the hydroxytyrosol glycosidic form has been identified in olives [25]. The crushing process is indeed a critical phase in olive oil processing, since a decrease of about half of the total phenolics can occur. Several modifications happen during this operation related to mechanical mixing, enzymatic and nonenzymatic hydrolyses and oxidations. The oleuropein content, a secoiridoid glucoside, decreases 94%, and the amount of other secoiridoid glucosides (demethyloleuropein and ligstroside) also decline. Glucosides can be biotransformed into their corresponding aglycones. First, the conversion to oleuropein or ligstroside aglycones occurs, followed by their decarboxymethylated forms. The crushing phase allowed an increase of hydroxytyrosol due to the degradation of compounds that contain hydroxytyrosol, which is also verified in tyrosol due to tyrosol-containing phenols hydrolysis [26]. 

Amongst all the samples, significant differences (*p* < 0.05) were only observed in the hydroxytyrosol content (Table 2). O1 (Alfândega-da-Fé) presented the highest amount (220 mg/100 g), followed by O3 (173 mg/100 g), O2 (122 mg/100 g) and O4 (63 mg/100 g).

Table 5 presents the chemical compositions regarding the total fat, fatty acids profile, total vitamin E and vitamers, protein and ash. The residual oil present in olive pomace depends on the olive cultivar, yield of extraction and the applied olive oil processing. Additionally, in this work, for sample preparation, a physical process was applied to remove the liquid phase, constituted from water and oil. Therefore, the initial characteristics of each pomace could also affect the oil amount of the extracts. 

The total fat contents are identical in three of the four samples (5 to 6% and 11% for O2). Likewise, the fatty acid present in a higher amount but with a similar value in the four samples (72 to 73%) was oleic acid (C18:1n9*cis*).

According to the scientific literature, fatty acids can exhibit antimicrobial activity depending on their structure—namely, the chain length, double bonds and action site. This bioaction is more relevant in Gram-positive than in Gram-negative bacteria, probably because of the Gram-positive outer membrane nonexistence, since fatty acids target the internal membrane. The antibacterial effect suggested to fatty acids is the inhibition of the cell’s nutrients uptake, peroxidation or auto-oxidation product formation (deleterious to cells), the disruption of the electron transport chain and leakage of the cell metabolites due to cell lysis [24].

The residual oil present in olive pomace that is passed to the extracts has a high content in oleic (C18:1n9*cis*) acid, followed by palmitic (C16:0) and linoleic (C18:2n6*cis*) acids, similar to the olive oil profile. The monounsaturated fatty acid oleic acid is, in fact, characteristic of olive oil. It is present in 73% in O1 and 72% in the other extracts in the study. The activity of membrane-associated enzymes can be inhibited by the oleic and linoleic acids. Specifically, unsaturated fatty acids, such as linoleic, can interfere in the bacteria fatty acid elongation process necessary to form the cellular materials [23]. O1 and O2 present the major contents in linoleic acid (9% and 11%, respectively). Overall, the samples had similar fatty acid profiles. In O1 and O3, which showed the best antimicrobial activity results, besides oleic and linoleic acid, heptadecanoic acid (C17:0) and *cis*-11-eicosenoic acid (C20:1n9) stand out in sample O1 and linolenic acid (C18:3n3) in O3. Different specific mechanisms for each fatty acid were explored, but they can be summed up in two key actions: the disruption of cell membranes and subsequent loss of cytoplasmic contents [23]. 

The vitamin E profile was determined in all the extracts (Table 5). Vitamin E is a blend of different liposoluble vitamers present in oils from vegetable sources such as olive oil. α-Tocopherol is the vitamer in higher amounts in all samples and the isoform biologically active with a potent antioxidant action [27]. Liposoluble compounds, such as α-tocopherol, can modify bacterial cell membranes, changing their fluidity and decreasing the barrier effect, allowing the passage of several compounds [22]. Table 6 resumes some mechanisms related to the α-tocopherol antimicrobial mechanism of action. Regarding the α-tocopherol contents in the samples, there are no significant differences among O1, O2 and O4, ranging from 1.7 to 2.0 mg/100 g of extract. O3 presented 0.8 mg/100 g. 

Considering the total fat amount (Table 5), O2 presented the highest amount of fat (10.5%) and a high content of α-tocopherol (1.8 mg/100 g). However, this sample had a weak antimicrobial activity (Table 4), suggesting once more that phenolics may be the main contributors to this biological action.

In general, regarding the incorporation, surface spreading and disk diffusion, the olive pomace functional ingredient acted against the studied bacteria (*S. aureus* and *E. coli*) at different levels, as shown in Table 4. The extracts’ MIC values ranged from 31.25 to 125 mg/mL (*S. aureus*) and from 62.5 to 125 mg/mL (*E. coli*).

*S. aureus* and *E. coli* are bacteria associated with foodborne diseases when the ingested food contains the pathogen or a toxin from their production [28]. *S. aureus* is widespread in the environment and is commensal on the human skin and mucosal membranes. It has been detected in several different foodstuffs, such as salads, milk, pork sausage, salmon steaks and shrimp, among others. *S. aureus* is one of the causes of infections most frequent. Hence, its presence in food is a public health concern [28,29]. On the other hand, *E. coli* is the main anaerobic facultative bacteria of the intestinal tract of humans and animals [30,31]. Some strains are more pathogenic to humans than others due to, for example, the ability to produce toxins. The foodborne diseases associated with *E. coli* are related to contaminated foodstuffs or water ingestion [28]. 

The cell wall structure and composition of Gram-positive and Gram-negative bacteria are different, which can explain the susceptibility variation towards plant extracts. Structurally, Gram-negative bacteria walls, such as *E. coli*, are more differentiated and complex than Gram-positive. Gram-negative bacteria have a stratified structure functionating as a wall. Its outer membrane lipopolysaccharide component makes them more resistant to antibacterial agents [20]. Gram-positive (*S. aureus*) bacteria have a homogeneous wall that does not contain phospholipids. Gram-positive bacteria are sensitive to several plant extracts. On the other hand, extracts with activity against Gram-negative bacteria are desired and beneficial for different applications [20]. 

In this study, none of the samples exhibited antimicrobial activity against *C. albicans*, an opportunistic and common fungal pathogen. It is usually present in the human gastrointestinal tract or is ingested with food. The disorders caused by this microorganism are more severe, as the host is compromised. It can be a health concern, for example, in antibacterial treatments with an unbalanced microbiota [32]. *C. albicans* is a morphological and physiologically highly variable and adaptable fungus [33]. 

Flavonoids, phenolic acids, polyenes, oligopeptides, terpenoids, macrolides and alkaloids are examples of compounds with described fungicide activity. Nevertheless, for olive leaf extracts, moderate antifungal activity was reported—in particular, for oleuropein [10,13,34]. Zorić et al. tested the effect of oleuropein on *C. albicans* cell membranes, showing that this compound affected the cell membrane [34]. Oleuropein is mainly found in olive leaves and fruits but scarcely in extra virgin olive oil and pomace owing to its hydrophilicity and susceptibility to degradation [35]. 

Nevertheless, hydroxytyrosol can represent ~50% of the total phenolics in extracts of olive pomace [5]. Medina-Martínez et al. reported that pure hydroxytyrosol solutions at different concentrations showed limited antimicrobial activity. Hence, the antimicrobial results observed can be due not just to the hydroxytyrosol content but, most likely, to a synergistic effect among all the phenolics and other compounds, such as the lipophilic ones present in the olive pomace extract [36]. 

The total protein and ash contents of the samples showed differences. The protein content was higher in O4 (4%) and O3 (3%), whereas the other samples presented 1%. Regarding the total minerals (ash), O4 was the richest sample (17%), while the other samples ranged between 10 and 11% (Table 5).

Considering the chemical profile of the samples and the variability and differences in the parameters analyzed, it seems that phenolics, particularly hydroxytyrosol, are the key compounds responsible for the antimicrobial activity described. Nevertheless, other components are possibly operating synergistically with phenolics against bacteria rather than individually. 

## 3. Materials and Methods

### 3.1. Chemicals and Reagents

Chemicals and reagents were of analytical grade and acquired from Merck (Darmstadt, Germany) (Kjeldahl tablets, sulfuric acid, absolute ethanol, sodium carbonate decahydrate, sodium hydroxide, n-hexane and anhydrous sodium sulfate) and Sigma-Aldrich (St. Louis, MO, USA) (Folin–Ciocalteu’s reagent, gallic acid, catechin, heptahydrate ferrous sulfate, 2,2-diphenyl-1-picrylhydrazyl radical, 2,2′-azino-bis(3-ethylenbenzothiazoline-6-sulfonic acid, Trolox, ferric chloride, 2,4,6-tripyridyltriazine, sodium nitrite, aluminum chloride, sodium acetate, hydroxytyrosol and Supelco 37 FAME Mix). Boric acid 4% was purchased from Panreac (Barcelona, Spain) and methanol from Honeywell International, Inc. (Morris Plains, NJ, USA). Sodium chloride and chloroform were acquired from VWR Chemicals (Alfragide, Portugal). The tocopherol and tocotrienol standards and tocol were obtained from Calbiochem (La Jolla, CA, USA) and Matreya Inc. (State College, PA, USA), respectively. The solvents (HPLC grade) were acquired from Chem-Lab (Zedelgem, Belgium) or Merck (Darmstadt, Germany). A Milli-Q water purification system (Millipore, Bedford, MA, USA) was used to treat and obtain the ultrapure water. 

### 3.2. Sampling and Preparation of the Olive Pomace Functional Ingredient

The olive pomaces were collected in two-phase olive mills in Portugal during the season 2017/2018. Two olive pomaces from Alfândega da Fé and Valpaços (northeast region) and two from Beja and Ferreira do Alentejo (south) were selected. Olive pomaces were constituted by a mixture of olive cultivars (Table 1). 

**Figure 1:**
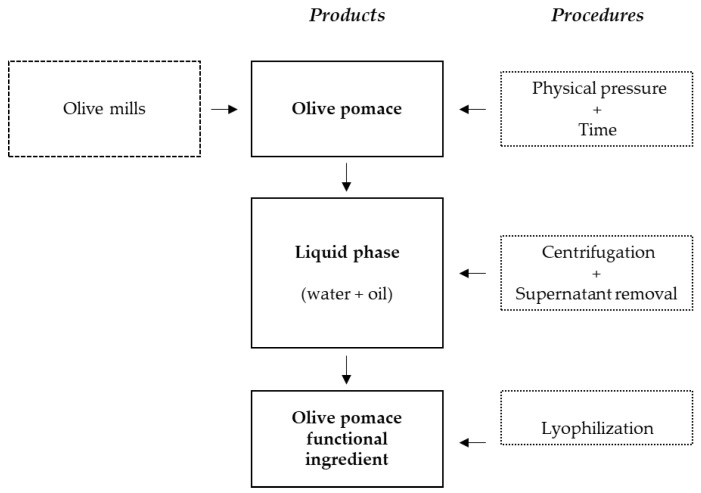
Obtention of the olive pomace functional ingredients.

After homogenization, each pomace (1 kg) was submitted to the process described in the patent international application PCT/IB2018/060111 (2019) [37] to obtain a functional ingredient. A pressing force (200–300 bar) for 30 min was applied to obtain a liquid phase constituted by water and oil. Then, it was centrifuged (5000 rpm; 20 min), and the superior layer separated and lyophilized. In this way, the olive pomace-based samples O1, O2 (from Alfândega da Fé and Valpaços, respectively), O3 and O4 (from Beja and Ferreira do Alentejo, correspondingly) were obtained (Figure 1).

The process was individually applied to each olive pomace sample (Alfândega da Fé, Valpaços, Beja and Ferreira do Alentejo), and the respective olive pomace ingredients (O1, O2, O3 and O4) were obtained.

### 3.3. Total Fat

The total fat was assessed using the Bligh & Dyer method with modifications, according to Manirakiza et al. [38]. The lipidic fraction was extracted with methanol and chloroform, vortexed and deionized water was added. The samples were vortexed again and centrifuged (2000 rpm; 10 min). The lower layer was transferred to a flask, and a second extraction was performed with 10% (*v*/*v*) methanol and chloroform, followed by vortexing. Both phases containing fat were mixed, the solvents evaporated and the residue dried (100 °C) until a constant weight. The results were presented as g/100 g.

### 3.4. Total Protein 

The total protein was determined by the Kjeldahl method based on standard methods (AOAC 928.08) [39], using 6.25 as the nitrogen conversion factor [40]. The results were presented as g/100 g.

### 3.5. Ash

The ash content was determined based on the standard methods (AOAC 920.153) (incineration at 500 °C) [39]. The results were expressed as g/100 g.

### 3.6. Phytochemicals and Antioxidant Activity Assessment

The lyophilized samples obtained in Section 3.2. were reconstituted with deionized water. Then, the solutions were vortexed to complete solubilization and centrifuged (5000 rpm; 5 min), followed by filtration throughout the syringe filters (pore size 0.45 μm). The phytochemicals (total phenolics and flavonoids) and antioxidant activity (ferric-reducing antioxidant power, 2,2-diphenyl-1-picrylhydrazyl radical and 2,2´-azinobis(3-ethylenbenzothiazoline-6-sulfonic acid) cation radical scavenging abilities were evaluated by spectrophotometric methods using a Synergy HT Microplate Reader (BioTek Instruments, Inc., Winooski, VT, USA). 

#### 3.6.1. Total Phenolics

The assessment of the phenolics content was performed according to Costa et al. [41] with modifications. Hence, 150 µL of Folin–Ciocalteu´s reagent (1:10) and 120 µL of Na_2_CO_3_ (7.5%, *m*/*v*) were added to 30 mL of diluted extracts and standards (gallic acid and hydroxytyrosol) in a microplate. The results were presented in g of gallic acid equivalents (GAE) and hydroxytyrosol equivalents (HE)/100 g (dry weight). For that, two calibration curves using gallic acid (5–100 mg/L); *r* = 0.9999) and hydroxytyrosol (2–80 mg/L; *r* = 0.9999), respectively, were prepared, and the absorbance was read at 765 nm. 

#### 3.6.2. Total Flavonoids 

For the total flavonoid evaluation, catechin was used as the standard (results expressed as g of catechin equivalents (CE)/100 g (dry weight). The standard curve was plotted (2.5–400 mg/L; *r* = 1), and the absorbance of the diluted samples was measured at 595 nm according to the method described by Costa et al. [41]. 

#### 3.6.3. Ferric-Reducing Antioxidant Power (FRAP) 

The ferric-reducing antioxidant power was determined using a calibration curve prepared with ferrous sulfate (50–600 µmol/L; *r* = 0.9999). The FRAP reagent (acetate buffer (0.3 M), TPTZ solution (10 mM) and ferric chloride (20 mM)) were freshly prepared and added (265 µL) to the diluted sample aliquots and standards (35 µL). The absorbance was read at 595 nm and the results expressed as g ferrous sulfate equivalents (FSE)/100 g [41]. 

#### 3.6.4. 2,2-Diphenyl-1-Picrylhydrazyl Radical (DPPH^•^) Scavenging Ability (DPPH)

The samples’ DPPH^•^ scavenging ability was evaluated as described by Costa et al. [41] by analyzing the kinetics of the reaction at 525 nm (intervals of 2 min). For that, 270 μL of a DPPH^•^ solution (6.0 × 10^−5^ mol/L in ethanol) were added to 30 μL of diluted extracts. The endpoint was established at 20 min. Trolox was used as a standard to prepare the calibration curve (5.6–101.2 mg/L; *r* = 0.9979). The scavenging ability was expressed as g of Trolox equivalents (TE)/100 g. 

#### 3.6.5. 2,2′-Azinobis(3-Ethylenbenzothiazoline-6-Sulfonic Acid) Cation Radical (ABTS^•+^) Scavenging Ability (ABTS)

The extracts’ scavenging ability against ABTS^•+^ was determined according to the methodology described by Seiquer et al. [42]. A calibration curve was prepared (0.01–0.1 mg/L; *r* = 0.9908) and absorbance read at 730 nm. The results were presented as g of Trolox equivalents (TE)/100 g.

### 3.7. Hydroxytyrosol Content Analysis by HPLC-DAD-FLD

Aliquots (1 mL) of the lyophilized extracts prepared with deionized water were centrifuged (13,000; 10 min) and filtered using syringe filters (0.45 μm) before injection into the HPLC-DAD-FLD system (Jasco, Tokyo, Japan). The system was composed of a LC-NetII/ADC hardware interface, a pump (Jasco PU-2089), an automatic sampler (Jasco AS-2057 Plus) and a multiwavelength diode array detector (Jasco MD-2018 Plus) coupled to a fluorescence detector (Jasco FP-2020 Plus, Jasco Co., Tokyo, Japan) and a column thermostat (Jasco CO-2060 Plus) (Jasco, Tokyo, Japan). Hydroxytyrosol was quantified by fluorescence and monitored (λ excitation = 280 nm; λ emission = 330 nm). The gradient elution program used was: 0 min, 5% B; 30 min, 25% B; 50 min, 75% B; 55 min, 100% B; 60 min, 100% B; 63 min, 5% B, being solvent (A) 1% acetic acid and (B) 100% methanol. A chromatographic column (Zorbax-SB-C18; 250 × 4.6 mm, 5 μm; Agilent Technologies, Amstelveen, The Netherlands) was used at 20 °C with a flow rate of 1 mL/min and an injection volume of 20 µL. The hydroxytyrosol content was expressed as g/100 g (dry weight). 

### 3.8. Vitamin E Profile by HPLC-DAD-FLD

After extraction of the lipid fraction and sample preparation (n-hexane and 50 μL of 0.1-mg/mL tocol as the internal standard) [43], the solution was injected into an HPLC-DAD-FLD system (Jasco, Tokyo, Japan) equipped with a multiwavelength diode array detector (MD-2015) and a FP-2020 fluorescence detector (Jasco, Tokyo, Japan) (excitation = 290 nm; emission = 330 nm). A normal phase Supelcosil™ LC-SI column (75 mm × 3.0 mm, 3.0 μm) (Supelco, Bellefonte, PA, USA) was used to separate the vitamin E vitamers [43]. The vitamer identification was based on the retention time (compared with the standards) and in its UV spectra. The results were expressed as g/100 g of extract.

### 3.9. Fatty Acids Profile by GC-FID

The samples’ oil was extracted as described by Alves et al. [43], and the fatty acid methyl esters (FAMEs) were obtained according to ISO 12966–2:2011 (cold derivatization) [44]. FAME separation was attained in a gas chromatograph (Shimadzu GC-2010 Plus, Tokyo, Japan) coupled to a split/splitless AOC-20i autoinjector (Shimadzu, Tokyo, Japan) and a flame ionization detector (Shimadzu, Tokyo, Japan). A CP-Sil 88 silica capillary column (50 m × 0.25 mm i.d., 0.20-μm film thickness) (Varian, Middelburg, The Netherlands) was used. The temperature program was the following: 120 °C for 5 min, increased to 160 °C at 2 °C/min, held for 15 min and increased to 220 °C at 2 °C/min. The injector and detector temperatures were 250 and 270 °C, respectively. The FAME retention times were compared with those obtained with a standard mixture (FAME 37, Supelco, Bellefonte, PA, USA). Each fatty acid was expressed as a relative percentage. 

### 3.10. pH

The pH was measured directly in the extracts with pH meter Basic 20+ (Crison, Barcelona, Spain). 

### 3.11. Antimicrobial Activity

Different agar-based methods were used to study the antimicrobial activity of the extracts (500 mg/mL). The minimal inhibitory concentration (MIC) was determined by the broth microdilution method of all the positive results obtained after screening. *Staphylococcus aureus* ATCC^®^ 25923, *Escherichia coli* ATCC^®^ 25922 and *Candida albicans* ATCC^®^ 10231 were used to evaluate the antimicrobial activity in the study. Before the antimicrobial activity experiments, the microorganisms were cultured twice in a nutritive agar, such as blood agar (Biomerieux, Marcy-l’Étoile, France). For the antimicrobial activity assays, cell suspensions of 0.5 Mac Farland (CS) were prepared (turbidity scale) in 1 mL of 0.85% NaCl solution (sterilized). Antimicrobial activity screening was performed on the agar through three different methods. A positive result was considered when the presence of a microorganism growth zone of inhibition was greater than the inhibition zone produced only by the solvent (in this case, ultrapure sterile water) [45,46]. 

#### 3.11.1. Incorporation Method 

A total of 1 mL of CS was added to a petri dish and 25 mL of melted Mueller–Hinton agar at 50 °C (MH, Liofilchem, Roseto degli Abruzzi, Italy) were added. Circular motions homogenized the mixture, and solidification occurred at room temperature. After solidification, 6-mm diameter cavities were made with sterile disposable tips, followed by cavity sealing by adding Muller–Hinton (10 μL) to the well bottom. After the bottom added agar was solidified, a total of 50 μL of solvent and extract were added to different wells. Plates were incubated at 37 °C for the bacteria (24 h) and yeast (48 h).

#### 3.11.2. Surface Spreading Method 

CS were spread on Muller–Hinton agar by surface scattering with a sterile swab. According to the procedure of Section 3.11.1., the agar wells were filled with the extract and solvent (ultrapure sterile water).

#### 3.11.3. Agar Diffusion Method 

Muller–Hinton agar plates were surface seeded using a swab with CS. The extract and solvent were added (20 µL) to different blank paper disks (sterile) and placed in the seeded agar.

#### 3.11.4. Microdilution Method

For the extracts that showed antimicrobial activity in the previously described methods, the solvents with the respective microorganisms, the extract with only TSB (Tryptic Soy Broth, Liofilchem, Roseto degli Abruzzi, Italy) and the extract with the microorganism under study were tested by the microdilution methodology in a 96-well plate, according to the previously described methodology [45]. 

In the first well of a 96-well plate, 100 µL of extract/solvent were pipetted, with 100 µL of previously diluted 1:150 in concentrated TSB (2×) of CS. After, in wells 2–12, TSB (50 µL) and CS (50 µL) diluted (1:150) in 1× TSB were added. Then, in the 1–11th wells, 100 µL were transferred from well to well. The 12th well, containing only TSB and CS, was used as the positive control of the assay. The plates were incubated at the same conditions of Section 3.11.1.

### 3.12. Statistical Analysis 

A statistical analysis was performed using IBM SPSS Statistics v.25 (IBM Corp., Armonk, NY, USA). The data were expressed as the mean ± standard deviation. A one-way ANOVA was used to evaluate the differences between the samples, followed by Tukey’s HSD to make pairwise comparisons between the means. The level of significance for all the hypothesis tests was 0.05.

## 4. Conclusions

From this work stands out the hydroxytyrosol content that has well-known and remarkable health effects. Hence, the olive pomace functional ingredient can be advantageous as a nutritional profile enhancer. The antibacterial activity determined that the samples can be a natural food preservative against tested Gram-positive and Gram-negative bacteria. As a lower MIC value indicates that less extract is required to inhibit the growth of the microorganism analyzed, O1 presented the highest potential among all the samples. Regarding the chemical profile, the four olive pomace functional ingredients studied presented similar profiles differing in the amount of fat, which was superior in O2, and protein and ash (higher in O4). 

Noteworthy, olive pomace extract can be considered an all-in-one and advantageous ingredient, since it presents a mixture of lipidic and hydrophilic bioactive compounds usually not present in other plant extracts.

The balance between environmental sustainability, low-cost and high-yield extraction procedures is challenging. In this research, the use of a green method allowed us to obtain natural, plant-based and chemical-free extracts with antimicrobial activity. Nevertheless, further studies regarding enhancing the phenolic contents in the extracts must be explored to improve the antimicrobial activity. Since some phenolics can be trapped in glycosidic junctions and pomace fibers, the implementation of, for example, a hydrolysis step before pressure can be advantageous.

## Figures and Tables

**Table 1:** Predominant olive varieties in the olive pomaces from the northeast region (Trás-Os-Montes) and south (Alentejo) of Portugal.

Portugal Regions	Olive Pomace Geographical Origin	Predominant Olive Varieties
Northeast region (Trás-Os-Montes)	Alfândega da Fé	Cobrançosa
		Madural
		Verdeal Transmontana
		Cordovil
	Valpaços	Madural
		Cordovil
South (Alentejo)	Beja	Cobrançosa
	Ferreira do Alentejo	Arbosana

**Table 2:** Total phenolics; flavonoids; hydroxytyrosol content and antioxidant activity (FRAP, DPPH and ABTS) of the olive pomace extracts (dry weight).

	Phytochemicals				Antioxidant Activity		
Samples	TPC		TF	Hydroxytyrosol	FRAP	DPPH	ABTS
	g GAE/100 g	g HE/100 g	g CE/100 g	mg/100 g	g FSE/100 g	g TE/100 g	g TE/100 g
O1	3.49 ± 0.28 ^a^	2.63 ± 0.29 ^a^	2.47 ± 0.29 ^b^	220 ± 20.57 ^a^	2.76 ± 0.52 ^a^	0.6 ± 0.07 ^b^	1.13 ± 0.07 ^a^
O2	3.05 ± 0.07 ^b^	2.34 ± 0.12 ^b^	2.29 ± 0.12 ^b^	122.39 ± 1.92 ^c^	2.46 ± 0.13 ^a^	0.96 ± 0.21 ^a^	0.85 ± 0.03 ^b^
O3	3.83 ± 0.2 ^a^	2.8 ± 0.11 ^a^	3.17 ± 0.11 ^a^	172.7 ± 2.09 ^b^	2.76 ± 0.16 ^a^	0.91 ± 0.05 ^a^	1.1 ± 0.1 ^a^
O4	3.06 ± 0.23 ^b^	2.23 ± 0.2 ^b^	1.96 ± 0.09 ^c^	63.33 ± 3.59 ^d^	2.65 ± 0.51 ^a^	0.68 ± 0.07 ^b^	0.8 ± 0.04 ^b^

O1, Alfândega-da-Fé; O2, Valpaços; O3, Beja; O4, Ferreira do Alentejo. TPC, total phenolics content; TF, total flavonoids; FRAP, ferric-reducing antioxidant power; DPPH, 2,2-diphenyl-1-picrylhydrazyl radical scavenging ability, ABTS, 2,2′-azino-bis(3-ethylenbenzothiazoline-6-sulfonic acid) radical scavenging ability; GAE, gallic acid equivalents; HE, hydroxytyrosol equivalents; CE, catechin equivalents; FSE, ferrous sulfate equivalents; TE, Trolox equivalents. The results are expressed as the mean ± standard deviation. Within each column, different letters represent significant differences between the samples at *p* < 0.05.

**Table 3:** General results of the antimicrobial activity of the olive pomace extracts.

			Olive Pomace Extracts			
Microorganism	Strain	Type	O1	O2	O3	O4
*Escherichia coli*	ATCC^®^ 25922	Gram-negative	+	+	+	+
*Staphylococcus aureus*	ATCC^®^ 25923	Gram-positive	+	+	+	+
*Candida albicans*	ATCC^®^ 10231	Yeast	−	−	−	−

O1, Alfândega-da-Fé; O2, Valpaços; O3, Beja; O4, Ferreira do Alentejo. (+), positive; (−), negative.

**Table 4:** Results of the antimicrobial activity against *E. coli* and *S. aureus* of the olive pomace extracts.

		O1		O2		O3		O4	
	Microorganism	*E. coli*	*S. aureus*	*E. coli*	*S. aureus*	*E. coli*	*S. aureus*	*E. coli*	*S. aureus*
Method	Incorporation (IZ mm)	10	15	10	13	12	14	10	13
	Surface spreading (IZ mm)	10	15	10	14	9	14	0	12
	Disk diffusion (IZ mm)	0	8	0	8	0	8	0	7
	MIC (mg/mL)	62.5	31.25	125.0	62.5	125.0	31.25	125	125

O1, Alfândega-da-Fé; O2, Valpaços; O3, Beja; O4, Ferreira do Alentejo. MIC, minimal inhibitory concentration; IZ, inhibition zone.

**Table 5:** Chemical analysis of the olive pomace ingredients (total fat, fatty acid and vitamin E profiles, total protein, ash and pH) (dry weight).

	O1	O2	O3	O4
Total fat (g/100 g)	5.55 ± 0.02 ^b^	10.54 ± 1.99 ^a^	4.62 ± 0.24 ^b^	5.86 ± 0.06 ^b^
Fatty acids (relative %)				
C16:0 (Palmitic)	11.77 ± 0.0 ^c^	11.59 ± 0.04 ^d^	13.22 ± 0.03 ^b^	14.35 ± 0.02 ^a^
C16:1 (Palmitoleic)	0.74 ± 0.01 ^c^	0.62 ± 0.02 ^d^	0.84 ± 0.05 ^b^	1.28 ± 0.01 ^a^
C17:0 (Heptadecanoic)	0.19 ± 0.00 ^a^	0.16 ± 0.00 ^b^	0.12 ± 0.01 ^d^	0.14 ± 0.00 ^c^
C18:0 (Stearic)	3.16 ± 0.01 ^c^	3.65 ± 0.01 ^a^	3.44 ± 0.02 ^b^	2.28 ± 0.01 ^d^
C18:1n9*cis* (Oleic)	72.94 ± 0.03 ^a^	71.7 ± 0.05 ^c^	71.82 ± 0.05 ^b^	71.78 ± 0.03 ^b^
C18:2n6*cis* (Linoleic)	9.44 ± 0.03 ^b^	10.48 ± 0.02 ^a^	8.75 ± 0.02 ^c^	8.4 ± 0.02 ^d^
C20:0 (Arachidic)	0.43 ± 0.01 ^b^	0.44 ± 0.02 ^ab^	0.45 ± 0.02 ^ab^	0.47 ± 0.01 ^a^
C18:3n3 (α-Linolenic)	0.83 ± 0.01 ^b^	0.9 ± 0.02 ^a^	0.9 ± 0.01 ^a^	0.72 ± 0.00 ^c^
C20:1n9 (*cis*-11-Eicosenoic)	0.28 ± 0.01 ^a^	0.25 ± 0.01 ^b^	0.22 ± 0.01 ^c^	0.3 ± 0.01 ^a^
C22:0 (Behenic)	0.16 ± 0.00 ^b^	0.15 ± 0.00 ^b^	0.16 ± 0.01 ^b^	0.21 ± 0.01 ^a^
C24:0 (Lignoceric)	0.06 ± 0.01 ^b^	0.06 ± 0.00 ^b^	0.07 ± 0.01 ^a^	0.08 ± 0.00 ^a^
Total vitamin E (mg/100 g)	1.83 ± 0.01 ^a^	1.99 ± 0.44 ^a^	0.87 ± 0.06 ^b^	2.25 ± 0.17 ^a^
α-Tocopherol	1.69 ± 0.01 ^a^	1.78 ± 0.42 ^a^	0.77 ± 0.06 ^b^	1.96 ± 0.15 ^a^
α-Tocotrienol	0.04 ± 0.00 ^c^	0.07 ± 0.00 ^b^	0.04 ± 0.00 ^c^	0.21 ± 0.01 ^a^
β-Tocopherol	0.03 ± 0.00 ^c^	0.05 ± 0.00 ^a^	0.02 ± 0.00 ^d^	0.04 ± 0.00 ^b^
γ-Tocopherol	0.07 ± 0.00 ^b^	0.09 ± 0.01 ^a^	0.04 ± 0.00 ^c^	0.04 ± 0.00 ^c^
Total protein (g/100 g)	0.88 ± 0.01 ^c^	0.88 ± 0.03 ^c^	3.16 ± 0.03 ^b^	4.44 ± 0.01 ^a^
Ash (g/100 g)	11.25 ± 0.65 ^b^	10.63 ± 1.16 ^b^	9.93 ± 0.30 ^b^	16.68 ± 0.18 ^a^
pH	5.23 ± 0.02 ^c^	5.36 ± 0.04 ^b^	5.26 ± 0.01 ^c^	5.63 ± 0.01 ^a^

O1, Alfândega-da-Fé; O2, Valpaços; O3, Beja; O4, Ferreira do Alentejo. The results are expressed as the mean ± standard deviation. Within each line, different letters represent significant differences between the samples at *p* < 0.05.

**Table 6:** Olive pomace bioactive compounds and proposed mechanisms of action.

Compounds	Proposed Mechanism	References
Phenolics	Disruption of the membranes structure and leakage of the cellular components Hydroxyl groups promote the delocalization of electrons, leading to the reduction of the gradient across membranes Reduction of the redox potential of the growth medium, leading to microbial growth constraints	[14,17,18]
Flavonoids	Inactivation of the microbial adhesion, enzymes and cell envelope transport proteins Disruption of microbial membranes (lipophilic flavonoids) Perforation and/or a reduction of the membrane fluidity Inhibition of nucleic acid synthesis, energy metabolism and cell membrane synthesis	[16,19]
Hydroxytyrosol	Capability of chelating transition metals, reducing the reactivity of iron and copper by forming an inert metal–ligand complex, which decreases the bioavailability for bacterial growth Reduction of intracellular ATP concentrations Cell membrane depolarization Reduction of the bacterial protein content	[20,21]
Vitamin E *(α-tocopherol)*	Damage in the cell membrane affecting the essential components for the integrity of the membrane (reduction in membrane potential and loss of ions, cytochrome C, proteins and radicals, followed by the collapse of proton pumps and decrease in ATP), increasing the membrane permeability Interaction with the lipid bilayer of the bacteria cell membrane modifying the respiratory chain and energy production Capacity of acting in the cell envelope resulting in an imbalance in the fluid mosaic nature of the bacterial membrane	[22]
Fatty acids	Disruption of the electron transport chain by binding to electron carriers Leakage of cell metabolites via cell lysis Inhibition of nutrient uptake Formation of peroxidation/auto-oxidation products resulting in cell deactivation	[23,24]

## Data Availability

The data presented in this study are available in the article.

## References

[1] Aguilar C.N., Ruiz H.A., Rios A.R., Chávez-González M., Sepúlveda L., Rodríguez-Jasso R.M., Loredo-Treviño A., Flores-Gallegos A.C., Govea-Salas M., Ascacio-Valdes J.A. (2019). Emerging strategies for the development of food industries. Bioengineered.

[2] Kumar K., Yadav A.N., Kumar V., Vyas P., Dhaliwal H.S. (2017). Food waste: A potential bioresource for extraction of nutraceuticals and bioactive compounds. Bioresour. Bioprocess.

[3] European Commission (2020). Olive Oil—Detailed Information on the Market Situation, Price Developments, Balance Sheets, Production and Trade. https://ec.europa.eu/info/food-farming-fisheries/farming/facts-and-figures/markets/prices/price-monitoring-sector/plant-products/olive-oil_en.

[4] Di Giovacchino L., Preziuso S.M., Di Serio M.G., Mucciarella M.R., Di Loreto G., Lanza B. (2017). Double extraction of olive oil in large oil mills of Southern Italy: Effects on extraction efficiency, oil quality, and economy of the process. Eur. J. Lipid Sci. Technol..

[5] Nunes M.A., Costa A.S.G., Bessada S., Santos J., Puga H., Alves R.C., Freitas V., Oliveira M.B.P.P. (2018). Olive pomace as a valuable source of bioactive compounds: A study regarding its lipid- and water-soluble components. Sci. Total Environ..

[6] Gullón P., Gullón B., Astray G., Carpena M., Fraga-Corral M., Prieto M.A., Simal-Gandara J. (2020). Valorization of by-products from olive oil industry and added-value applications for innovative eco-foods. Food Res. Int..

[7] Ng K.R., Lyu X., Mark R., Chen W.N. (2019). Antimicrobial and antioxidant activities of phenolic metabolites from flavonoid-producing yeast: Potential as natural food preservatives. Food Chem..

[8] de Carvalho B.L., Salgueiro M.d.F., Rita P. (2015). Consumer sustainability consciousness: A five dimensional construct. Ecol. Indic..

[9] Olszewska M.A., Gędas A., Simões M. (2020). Antimicrobial polyphenol-rich extracts: Applications and limitations in the food industry. Food Res. Int..

[10] Korukluoglu M., Sahan Y., Yigit A. (2008). Antifungal properties of olive leaf extracts and their phenolic compounds. J. Food Saf..

[11] Gyawali R., Ibrahim S.A. (2014). Natural products as antimicrobial agents. Food Control.

[12] Mbaveng A.T., Sandjo L.P., Tankeo S.B., Ndifor A.R., Pantaleon A., Nagdjui B.T., Kuete V. (2015). Antibacterial activity of nineteen selected natural products against multi-drug resistant Gram-negative phenotypes. SpringerPlus.

[13] Diallinas G., Rafailidou N., Kalpaktsi I., Komianou A.C., Tsouvali V., Zantza I., Mikros E., Skaltsounis A.L., Kostakis I.K. (2018). Hydroxytyrosol (HT) analogs act as potent antifungals by direct disruption of the fungal cell membrane. Front. Microbiol..

[14] Xue J., Davidson P.M., Zhong Q. (2013). Thymol nanoemulsified by whey protein-maltodextrin conjugates: The enhanced emulsifying capacity and antilisterial properties in milk by propylene glycol. J. Agric. Food Chem..

[15] Peralbo-Molina Á., Priego-Capote F., de Castro M.D.L. (2012). Tentative identification of phenolic compounds in olive pomace extracts using liquid chromatography-tandem mass spectrometry with a quadrupole-quadrupole-time-of-flight mass detector. J. Agric. Food Chem..

[16] Cushnie T.P.T., Lamb A.J. (2011). Recent advances in understanding the antibacterial properties of flavonoids. Int. J. Antimicrob. Agents.

[17] Cueva C., Moreno-Arribas M.V., Martín-Álvarez P.J., Bills G., Vicente M.F., Basilio A., Rivas C.L., Requena T., Rodríguez J.M., Bartolomé B. (2010). Antimicrobial activity of phenolic acids against commensal, probiotic and pathogenic bacteria. Res. Microbiol..

[18] Stojković D., Petrović J., Soković M., Glamočlija J., Kukić-Marković J., Petrović S. (2013). In situ antioxidant and antimicrobial activities of naturally occurring caffeic acid, *p*-coumaric acid and rutin, using food systems. J. Sci Food Agric..

[19] Alghazeer R., Elmansori A., Sidati M., Gammoudi F., Azwai S., Naas H., Garbaj A., Eldaghayes I. (2017). In vitro antibacterial activity of flavonoid extracts of two selected Libyan algae against multi-drug resistant bacteria isolated from food products. J Biosci. Med..

[20] Yangui T., Dhouib A., Rhouma A., Sayadi S. (2009). Potential of hydroxytyrosol-rich composition from olive mill wastewater as a natural disinfectant and its effect on seeds vigour response. Food Chem..

[21] Guo L., Gong S., Wang Y., Sun Q., Duo K., Fei P. (2020). Antibacterial activity of olive oil polyphenol extract against *Salmonella Typhimurium* and *Staphylococcus aureus*: Possible mechanisms. Foodborne Pathog. Dis..

[22] Andrade J.C., Morais-Braga M.F.B., Guedes G.M.M., Tintino S.R., Freitas M.A., Menezes I.R.A., Coutinho H.D.M. (2014). Enhancement of the antibiotic activity of aminoglycosides by alpha-tocopherol and other cholesterol derivates. Biomed. Pharmacother..

[23] Yoon B.K., Jackman J.A., Valle-González E.R., Cho N.-J. (2018). Antibacterial free fatty acids and monoglycerides: Biological activities, experimental testing, and therapeutic applications. Int. J. Mol. Sci..

[24] Zhou J., Velliou E., Hong S.H. (2020). Investigating the effects of nisin and free fatty acid combined treatment on *Listeria monocytogenes* inactivation. LWT-Food Sci. Technol..

[25] Klen T.J., Vodopivec B.M. (2012). The fate of olive fruit phenols during commercial olive oil processing: Traditional press *versus* continuous two- and three-phase centrifuge. LWT-Food Sci. Technol..

[26] Klen T.J., Wondra A.G., Vrhovšek U., Sivilotti P., Vodopivec B.M. (2015). Olive fruit phenols transfer, transformation, and partition trail during laboratory-scale olive oil processing. J. Agric. Food Chem..

[27] Tintino S.R., Morais-Tintino C.D., Campina F.F., Pereira R.L., Costa M.d.S., Braga M.F.B.M., Limaverde P.W., Andrade J.C., Siqueira-Junior J.P., Coutinho H.D.M. (2016). Action of cholecalciferol and alpha-tocopherol on *Staphylococcus aureus* efflux pumps. EXCLI J..

[28] Bintsis T. (2017). Foodborne pathogens. AIMS Microbiol..

[29] Denayer S., Delbrassinne L., Nia Y., Botteldoorn N. (2017). Foodborne outbreak investigation and molecular typing: High diversity of *Staphylococcus aureus* strains and importance of toxin detection. Toxins.

[30] Palmeira J.D., Haenni M., Metayer V., Madec J.-Y., Ferreira H.M.N. (2020). Epidemic spread of IncI1/pST113 plasmid carrying the Extended-Spectrum Beta-Lactamase (ESBL) blaCTX-M-8 gene in *Escherichia coli* of Brazilian cattle. Vet. Microbiol.

[31] Mota R., Pinto M., Palmeira J., Gonçalves D., Ferreira H. (2020). Multidrug-resistant bacteria as intestinal colonizers and evolution of intestinal colonization in healthy university students in Portugal. Access Microbiol..

[32] Rajkowska K., Kunicka-Styczyńska A. (2018). Typing and virulence factors of foodborne *Candida* spp. isolates. Int. J. Food Microbiol..

[33] da Silva Dantas A., Lee K.K., Raziunaite I., Schaefer K., Wagener J., Yadav B., Gow N.A.R. (2016). Cell biology of *Candida albicans*–host interactions. Curr. Opin. Microbiol..

[34] Zorić N., Kopjar N., Bobnjarić I., Horvat I., Tomić S., Kosalec I. (2016). Antifungal activity of oleuropein against *Candida albicans*—The in vitro study. Molecules.

[35] Li X., Liu Y., Jia Q., LaMacchia V., O’Donoghue K., Huang Z. (2016). A systems biology approach to investigate the antimicrobial activity of oleuropein. J. Ind. Microbiol. Biotechnol..

[36] Medina-Martínez M.S., Truchado P., Castro-Ibáñez I., Allende A. (2016). Antimicrobial activity of hydroxytyrosol: A current controversy. Biosci. Biotechnol. Biochem..

[37] Nunes M.A., Costa. A.S.G., Oliveira M.B.P.P., Applicant: University of Porto (2019). Foodstuff Composition Comprising a Derivate of Olive Pomace.

[38] Manirakiza P., Covaci A., Schepens P. (2001). Comparative study on total lipid determination using Soxhlet, Roese-Gottlieb, Bligh & Dyer, and modified Bligh & Dyer extraction methods. J. Food Compost. Anal..

[39] (2012). Official Methods of Analysis.

[40] Tontisirin K. (2003). Chapter 2: Methods of food analysis. Food Energy: Methods of Analysis and Conversion Factors: Report of a Technical Workshop.

[41] Costa A.S.G., Alves R.C., Vinha A.F., Costa E., Costa C.S.G., Nunes M.A., Almeida A.A., Santos-Silva A., Oliveira M.B.P.P. (2018). Nutritional, chemical and antioxidant/pro-oxidant profiles of silverskin, a coffee roasting by-product. Food Chem..

[42] Seiquer I., Rueda A., Olalla M., Cabrera-Vique C. (2015). Assessing the bioavailability of polyphenols and antioxidant properties of extra virgin argan oil by simulated digestion and Caco-2 cell assays. Comparative study with extra virgin olive oil. Food Chem..

[43] Alves R.C., Casal S., Oliveira M.B.P.P. (2009). Determination of vitamin E in coffee beans by HPLC using a micro-extraction method. Food Sci. Technol. Int..

[44] ISO 12966-2:2011—Animal and vegetable fats and oils—Gas chromatography of fatty acid methyl esters—Part 2: Preparation of methyl esters of fatty acids.

[45] Almeida D., Pinto D., Santos J., Vinha A.F., Palmeira J., Ferreira H.N., Rodrigues F., Oliveira M.B.P.P. (2018). Hardy kiwifruit leaves (*Actinidia arguta*): An extraordinary source of value-added compounds for food industry. Food Chem..

[46] Palmeira J.D., Ferreira S.B., de Souza J.H., de Almeida J.M., Figueiredo M.C., Pequeno A.S., Arruda T.A., Antunes R.M.P., Catão R.M.R. (2010). Evaluation of the antimicrobial activity in vitro and determination of minimal the inhibitory concentration of hidroalcoholicoc extracts of angico in strains *Staphylococcus aureus*. Br. J. Clin. Anal..

